# Molecular detection and zoonotic genotyping of *Enterocytozoon bieneusi* in Siberian tigers (*Panthera tigris altaica*), Northeast China

**DOI:** 10.1007/s00436-026-08715-0

**Published:** 2026-06-16

**Authors:** Hang Tian, Zhiying Cui, Zhiyou Lv, Mengqiu Liu, Yang Liu, Yilin Zhao, Weiyan Yin, Mengru Zhao, Hongyan Yu, Mengchao Zhou, Yaxian Lu, Dapeng Li, Ming Gong, Haitao Xu, Zhijun Hou

**Affiliations:** 1 Laboratory of Vector-Borne Diseases and Pathogens Ecology, College of Wildlife and Protected Area, Northeast Forestry University, Harbin, 150040 People’s Republic of China; 2 Siberian Tiger Park, Harbin, 150028 People’s Republic of China

**Keywords:** *Enterocytozoon bieneusi*, Genotype, ITS, *Panthera tigris altaica*, China

## Abstract

*Enterocytozoon bieneusi* is an important zoonotic pathogen. This study examined its prevalence and genotypes in Siberian tigers in Northeast China. Fecal samples from 97 captive and 5 wild tigers were analyzed by nested PCR targeting the ITS region. Prevalence in captive tigers was 40.2% (39/97), with no significant site differences. Three genotypes were identified: D (89.7%), Peru8 (7.7%), and Type IV (2.6%), all within zoonotic Group 1. *E. bieneusi* was detected for the first time in a wild Siberian tiger (genotype D). The predominance of genotype D—common in poultry—suggests dietary transmission through contaminated chicken meat. These results indicate high prevalence of zoonotic genotypes in tigers, underscoring cross-species transmission risks and the need for strengthened biosecurity and surveillance.

**Supplementary Information:**

The online version contains supplementary material available at 10.1007/s00436-026-08715-0.

## Introduction

The Siberian tiger (*Panthera tigris altaica*) is a critically endangered species. Infectious disease management is crucial for its conservation (Wang et al. 2015). *Enterocytozoon bieneusi*, a common microsporidian pathogen, infects humans and a wide range of animals globally (Li and Xiao 2021). Over 500 genotypes have been identified, classified into at least 15 phylogenetic groups (Jiang et al. 2024), updating the earlier 11-group framework. Genotypes in Group 1 have broad host ranges and significant zoonotic potential (Li et al. 2019).

As apex predators, Siberian tigers may serve as underestimated reservoirs for zoonotic pathogens like *E. bieneusi*. In captive settings, close proximity between felids and handlers poses risks for direct occupational exposure (He et al. 2018). Conversely, free-ranging tigers can shed environmentally resilient spores into soil and water, creating indirect cross-species transmission risks for sympatric wildlife, livestock, and local human communities (Figueiredo et al. 2023). Therefore, identifying the circulating genotypes in tigers is essential to assess their implications for wildlife conservation and public health.

Although *E. bieneusi* has been detected in various wildlife (Amer et al. 2019; Zhang et al. 2022), data on Siberian tigers are restricted to a single captive case of genotype D in China (Li et al. 2015). To address the lack of comprehensive and comparative data between captive and wild populations, this study investigated the prevalence, genetic diversity, and clinical status (assessed via stool consistency) of *E. bieneusi* in both captive and wild Siberian tigers in Northeast China.

## Materials and methods

### Ethics approval

This study relied exclusively on non-invasive fecal sampling without animal disturbance. All protocols were approved by the Institutional Animal Care and Use Committee of Northeast Forestry University and authorized by the Siberian Tiger Park.

### Sample collection and DNA extraction

**Fig. 1:**
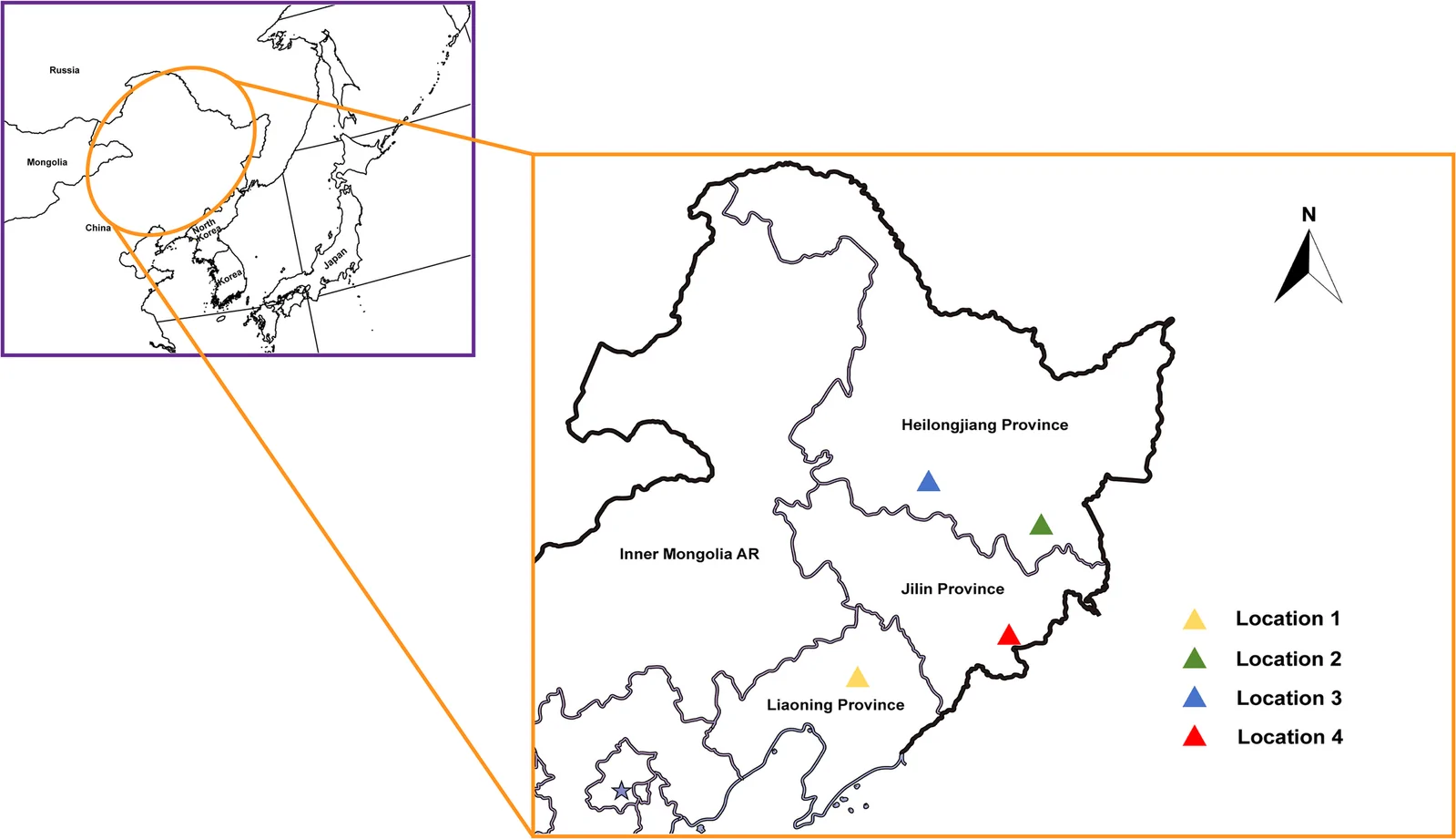
Specific locations where samples were collected in this study. ▲ Sampling points

During 2024–2025, 97 fresh fecal samples were collected from the enclosure floors of three captive Siberian tiger sanctuaries in Northeast China (Locations 1–3, Fig. 1), and 5 from the forest ground of wild tigers in the Changbai Mountain region (Location 4, Fig. 1). To minimize environmental contamination, only the interior portion of fresh fecal deposits was sampled using sterile spatulas. At Locations 1–3, enclosures were dedicated solely to Siberian tigers and isolated from other large carnivores; poultry used as feed were housed separately. All samples were stored at -20 ℃ until DNA extraction. Fecal morphology was classified using the Bristol Stool Chart (Harvey et al. 2023) as hard (Types 1–2), normal (Types 3–5), or loose (Types 6–7). Statistical analyses were performed via SPSS using Fisher’s exact test to evaluate associations between *E. bieneusi* infection status, stool consistency, and collection sites. Statistical significance was defined at *p* < 0.05.

### Molecular detection and genotyping

*E. bieneusi* was detected via nested PCR targeting a 390 bp fragment of the ribosomal internal transcribed spacer (ITS) region (Zhang et al. 2022). The primary PCR utilized outer primers EBITS3 (5′-GGTCATAGGGATGAAGAG-3′) and EBITS4 (5′-TTCGAGTTCTTTCGCGCTC-3′), while the secondary round utilized inner primers EBITS1 (5′-GCTCTGAATATCTATGGCT-3′) and EBITS2.4 (5′-ATCGCCGACGGATCCAAGTG-3′). Each 25µL reaction mixture contained 12.5 µL Taq DNA polymerase, 1 µL of each primer, and 2 µL of template DNA. Cycling conditions for both rounds comprised 35 cycles of denaturation at 94 °C for 45 s, extension at 72 °C for 60 s, and a final extension at 72 °C for 10 min, with annealing temperatures optimized at 57 °C (primary) and 55 °C (secondary) for 45 s. Amplicons were visualized via 2% agarose gel electrophoresis.

Positive secondary PCR products were sequenced commercially (Takara Bio Inc., Harbin). Sequences were aligned and compared with reference sequences from GenBank using MEGA 11 software to determine genotypes.

### Phylogenetic analysis

Phylogenetic analysis was performed in MEGA 11 using sequences aligned via ClustalW. A neighbor-joining tree was constructed based on the Kimura 2-parameter model, with node support evaluated by 1,000 bootstrap replicates (values > 50% shown). Representative sequences of each unique genotype variant from captive (PP968731-PP968739) and wild (PX579110) tigers were analyzed alongside GenBank reference sequences.

## Results

### Prevalence of *E. bieneusi*

The overall prevalence in captive Siberian tigers was 40.2% (39/97). Infection rates at Locations 1, 2, and 3 were 35.1% (13/37), 35.5% (11/31), and 51.7% (15/29), respectively, with no significant differences (*p* > 0.05). One of the five wild tiger samples tested positive (20.0%).

### *E. bieneusi* genotypes and phylogenetic relationship

**Fig. 2:**
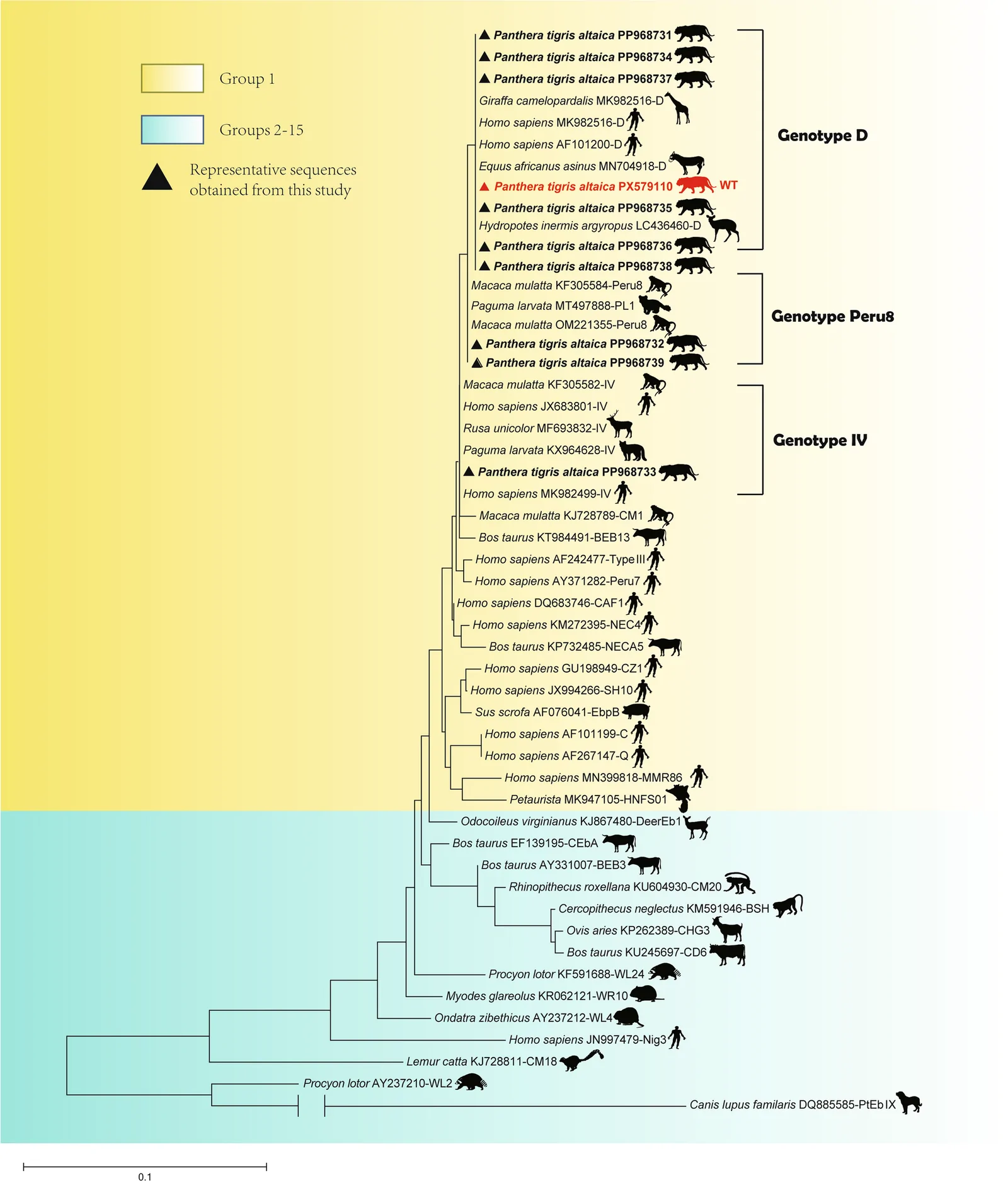
The phylogenetic tree was constructed based on the neighbor-joining analysis of ITS sequences. Bootstrap values > 50% from 1,000 replicates are indicated on the nodes. Silhouettes of animals used in the phylogenetic tree are sourced from PhyloPic (www.phylopic.org). ▲: sequences from captive Siberian tigers; ▲: the sequence from the wild Siberian tiger. Phylogenetic groups are defined according to the updated classification of Jiang et al. (2024)

All 40 positive samples yielded three known *E. bieneusi* genotypes: D (89.7%; 35 captive, 1 wild), Peru8 (7.7%; 3 captive), and Type IV (2.6%; 1 captive). Notably, the wild tiger sequence represents the first detection of *E. bieneusi* in a free-ranging Siberian tiger. Phylogenetic analysis of 10 representative sequences alongside GenBank references confirmed that all identified genotypes (D, Peru8, and Type IV) cluster within the zoonotic Group 1 (Fig. 2).

### *E. bieneusi* detection and association with stool consistency

Stool consistency profiles (hard, normal, and loose) showed no significant correlation with *E. bieneusi* infection status (*p* > 0.05). Despite the high prevalence, the absence of overt clinical symptoms suggests these tigers are likely subclinical carriers. However, because these DNA-positive outcomes solely reflect the presence of parasitic genetic material, they must be interpreted with caution as they do not definitively confirm active, replicating infections within the hosts.

## Discussion

The high *E. bieneusi* prevalence (40.2%) observed in captive Siberian tigers in Northeast China extends the initial report by Li et al. (2015), who first identified this pathogen in a captive Siberian tiger in a Chinese zoo. The dominant zoonotic genotypes (D, Peru8, Type IV) (Li et al. 2019; Chabchoub et al. 2012) indicate a public health risk. Although direct physical contact between the public and Siberian tigers is non-existent or strictly controlled, these zoonotic genotypes pose a potential public health risk through indirect transmission routes. For instance, spores shed in tiger feces can contaminate the surrounding soil and water systems via surface runoff, potentially entering the local food or water supply. Furthermore, occupational exposure remains a concern for animal keepers, veterinarians, and staff at tiger sanctuaries, who may act as intermediaries for pathogen dispersal. Thus, the presence of these genotypes in tigers highlights the importance of environmental monitoring and biosafety protocols in and around tiger habitats. Critically, genotype D was detected in a wild Siberian tiger for the first time. Despite geographical isolation, the shared genotype D suggests indirect pathogen circulation between captive and wild tiger landscapes. This transmission may be mediated by environmental matrices or highly mobile intermediate hosts and prey (e.g., rodents or birds) traversing the captive-wild interface (Galván et al. 2013), highlighting a potential spillover risk. Given that genotype D is a well-known generalist common in poultry (Wang et al. 2018; da Cunha et al. 2016), the tigers’ chicken-based diet (He et al. 2018) represents a plausible transmission pathway, consistent with findings in other captive carnivores (Figueiredo et al. 2023) and underscoring the need for dietary biosecurity screening.

Currently, direct documentation regarding the clinical or pathological effects of *E. bieneusi* on Siberian tigers remains entirely absent. Although our stool consistency analysis indicates a predominantly subclinical carrier status, the pathogen’s nature as an obligate intracellular parasite causing enterocyte destruction (Li and Xiao 2021) should not be ignored. Such opportunistic colonization may pose insidious health risks—including chronic metabolic deficiencies or diarrhea—to vulnerable individuals (e.g., cubs, senescent animals, or pregnant females) or during periods of severe captive and environmental stress.

Captive conditions, such as high animal density, likely facilitate pathogen transmission (Zhu et al. 2022; Norte et al. 2022). This observed subclinical carrier status may be driven by host immunocompetence (Chu et al. 2024), low parasite burdens, or genotype-specific virulence traits. Furthermore, while these tigers undergo routine veterinary screenings, the confounding effects of co-infections with other viruses, bacteria, or parasites remain unexamined. Comprehensive multi-pathogen panels and host immune response evaluations are warranted in future studies to clarify the precise mechanisms underlying these asymptomatic microsporidian infections.

Stool consistency (Hard: *P* = 13, *N* = 18; Normal: *P* = 7, *N* = 18; Loose: *P* = 19, *N* = 22) showed no significant association with *E. bieneusi* infection (*p* > 0.05), confirming that these tigers act as asymptomatic reservoirs.

A primary limitation is the exclusive reliance on fecal DNA detection, which cannot definitively differentiate active infections from environmental contamination or the transient mechanical passage of residual material from poultry-derived feed. Because invasive diagnostics like histopathology or antigen testing are restricted due to the endangered status of Siberian tigers, true colonization remains challenging to define, necessitating cautious interpretation of the prevalence data. Future research utilizing multi-locus sequence typing (MLST) or whole-genome sequencing, combined with direct screening of feed and environmental water, is required to distinguish true ongoing infections from passive passage.

## Conclusion

This study reveals a high prevalence of zoonotic *E. bieneusi* in captive Siberian tigers, with genotype D predominating. Its first detection in a wild tiger suggests potential spillover from captive settings. Given that genotype D is common in poultry, these findings implicate dietary transmission via contaminated chicken meat as a plausible pathway.

To mitigate transmission risks, we propose establishing routine molecular monitoring for both captive tigers and handlers to track zoonotic spillover from subclinical carriers. Biosecurity within sanctuaries should be optimized by screening or treating poultry-derived feed to prevent dietary exposure to genotype D, alongside enforcing stringent waste management and disinfection to block fecal spore dissemination into water and soil. Finally, microsporidian screening should be systematically integrated into pre-release health protocols for wild reintroduction programs to prevent pathogen spillover across the captive-wild interface, thereby safeguarding public health and fragile ecosystems.

## Supplementary Information

Supplementary Material 1.

## Data Availability

No datasets were generated or analysed during the current study.
