# Supplementary material for: Molecular detection and zoonotic genotyping of Enterocytozoon bieneusi in Siberian tigers (Panthera tigris altaica), Northeast China
Source: Parasitol Res. 2026 Jun 16;125(1):103. doi: 10.1007/s00436-026-08715-0 (PMC13506582; doi:10.1007/s00436-026-08715-0)
Supplement: Supplementary file 1 [file 436_2026_8715_MOESM1_ESM.pdf]

Supplementary File: Documentation of Molecular Validation for *Enterocytozoon bieneusi* Detection

1. Statement Regarding Raw Gel Electrophoresis Images

We sincerely apologize that the original uncropped gel electrophoresis images corresponding to the preliminary PCR screening are unavailable for submission. During the early molecular screening phase of this study (2024–2025), some gel image files were unfortunately not fully retained following completion of the initial experiments.

We recognize the importance of preserving original experimental records and have since strengthened our laboratory data management and archival procedures for all ongoing and future studies.

Importantly, the absence of the original gel photographs does not affect the authenticity of the sequencing results or the conclusions of the present study.

2. Alternative Validation via Sanger Sequencing and GenBank Deposition

All PCR-positive samples included in this study were further confirmed by commercial bidirectional Sanger sequencing (Takara Bio Inc., Harbin, China) targeting the approximately 390 bp internal transcribed spacer (ITS) region of Enterocytozoon bieneusi.

Genotype identification was determined based on nucleotide sequence alignment and phylogenetic comparison using MEGA 11 software, rather than solely on visualization of PCR bands.

The following genotypes were confirmed:

- Genotype D
- Genotype Peru8
- Genotype Type IV

3. Publicly Accessible Sequence Data

To ensure full transparency and independent verification, all representative nucleotide sequences obtained from both captive and wild Siberian tigers have been deposited in the NCBI GenBank database under the following accession numbers:

Population	GenBank Accession Numbers
Captive Siberian tigers	PP968731, PP968732, PP968733, PP968734, PP968735, PP968736, PP968737, PP968738, PP968739
Wild Siberian tiger	PX579110

4. Full Sequence Information (All Accessions)

Below are all nucleotide sequences deposited in GenBank for this study:

Captive populations:

>PP968731
TGTAGGAGCTTCGGCTCTGAATATCTATGGCTAGATAAAGTACAAGTCGTAACAAGGTT
TCAGTTGGAGAACCAGCTGAAGGATCATTTTCAGTTTTTGGGGTGTGGGTATCGGAAT
GTGTGGTAGGTGATGTGTGTGTGTATGGGGGATGCCGAGGGGACCAGCGGTGCGGTGG
TGTGTGCAGGCGTGAGAGTGTATCTGCAAGTGTGAGGGATGTGGGTGCAGCGAGTTAG

AGGTGGTTCCATGTGGAATAGTGGGATTGGTACGTGATGGTTGGATGGGGGAATGATGT
GTGTATGGGTGAGGAAAATCGGAGGTTGCGGTGCGAGCGGCAGTAGGGTGCCCTCAA
AAGGTGTATTTCGAAATATCCCTAAT
>PP968732
CGGCTCTGAATATCTATGGCTAGATAAAGTACAAGTCGTAACAAGGTTTCAGTTGGAGA
ACCAGCTGAAGGATCATTTTCAGTTTTTGGGGTGTGGGTATCGGAATGTGTGGTAGGTG
ATGTGTGTGTGTATGGGGGATGCCGAGGGGACCAGCGGTGCGGTGGTGTGTGCAGGC
GTGAGAGTGTATCTGCAAGGGTGAGGGATGTGGGTGCAGCGAGTTAGAGGTGGTTCCA
TGTGGAATAGTGGGATTGGTACGTGATGGTTGGATGGGGGAATGATGTGTGTATGGGTG
AGGAAAATCGGAGGTTGCGGTGCGAGCGGCAGTAGGGTGCCATCAA
>PP968733
GAAGAGCTTCGGCTCTGAATATCTATGGCTAGATAAAGTACAAGTCGTAACAAGGTTTC
AGTTGGAGAACCAGCTGAAGGATCATTTTCAGTTTTTGGGGTGTGGGTATCGGAATGT
GTGGTAGGTGATGTGTGTGTGTATGGGGGATGCCGAGGGGACCAGCGGTGCGGTGGTG
TGTGTAGGCGTGAGAGTGTATCTGCAAGGGTGAGGGATGTGGGTGCAGCGAGTTAGA
GGTGGTTCCATGTGGAATAGTGGGATTGGTACGTGATGGTTGGATGGGGGAATGATGTG
TGTATGGGTGAGGAAAATCGGAGGTTGCGGTGCGAGCGGCAG
>PP968734
GGCTCTGAATATCTATGGCTAGATAAAGTACAAGTCGTAATAAGGTTTCAGTTGGAGAA
CCAGCTGAAGGATCATTTTCAGTTTTTGGGGTGTGGGTATCGGAATGTGTGGTAGGAGA
TGTGTGTGTGTATGGGGGATGCCGAGGGGACCAGCGGTGCGGTGGTGCGTGCAGGCG
TGAGAGTGTATCTGCAAGTGTGAGGGATGTGGGTGCAGCGAGTTAGAGGTGGTTCCAT
GTGGAATAGTGGGATTGGTACGTGATGGTTGGATGGGGGAATGATGTGTGTATGGGTGA
GGAAAATCGGAGGTTGCGGTGCGAGCGGCAGTA
>PP968735
GCTTCGGCTCTGAATATCTATGGCTAGATAAAGTACAAGTCGTAACAAGGTTTCAGTTG
GAGAACCAGCTGAAGGATCATTTTCAGTTTTTGGGGTGTGGGTATCGGAATGTGTGGT
AGGTGATGTGTGTGTGTATGGGGGATGCCGAGGGGACCAGCGGTGCGGTGGTGTGTGC
AGGCGTGAGAGTGTATCTGCAAGTGTGAGGGATGTGGGCGCAGCGAGTTAGAGGTGG
TTCCATGTGGAATAGTGGGATTGGTACGTGATGGTTGGATGGGGGAATGATGTGTGTAT
GGGTGAGGAAAATCGGAGGTTGCGGTGCGAGCGGCAGTAG
>PP968736
GCTTCGGCTCTGAATATCTATGGCTAGATAAAGTACAAGTCGTAACAAGGTTTCAGTAG
GAGAACCAGCTGAAGGATCATTTTCAGTTTTTGGGGTGTGGGTATCGGAATGTGTGGT
AGGTGATGTGTGTGTGTATGGGGGATGCCGAGGGGACCAGCGGTGCGGTGGTGTGTGC
AGGCGTGAGAGTGTATCTGCAAGTGTGAGGGATGTGGGTGCAGCGAGTTAGAGGTGG
TTCCATGTGGAATAGTGGGATTGGTACGTGATGGTTGGATGGGGGAATGATGTGTGTAT
GGGTGAGGAAAATCGGAGGTTGCGGTGCGAGCGGCAG
>PP968737
TGAAGAGCTTCGGCTCTGAATATCTATGGCTAGATAAAGTACAAGTCGTAACAAGGGTT
CAGTTGGAGAACCAGCTGAAGGATCATTTTCAGTTTTTGGGGTGTGGGTATCGGAATG
TGTGGTAGGTGATGTGTGTGTGTATGGGGGATGCCGAGGGGACCAGCGGTGCGGTGGT
GTGTGCAGGCGTGAGAGTGTATCTGCAAGTGTGAGGGATGTGGGTGCAGCGAGTTAG
AGGTGGTTCCATGTGGAATAGTGGGATTGGTACGTGATGGTTGGATGGGGGAATGATGT

GTGTATGGGTGAGGAAAATCGGAGGTTGCGGTGCGAGCGGCAGT
>PP968738
TGAAGAGCTTCGGCTCTGAATATCTATGGCTAGATAAAGTACAAGTCGTAACAAGGTTT
CAGTTGGAGAACCAGCTGAAGGATCATTTTCAGTTTTTGGGGTGTGGGTATCGGAATG
TGTGGTAGGTGGTGTGTGTGTGTATGGGGGATGCCGAGGGGACCAGCGGTGCGGTGGT
GTGTGCAGGCGTGAGAGTGTATCTGCAAGTGTGAGGGATGTGGGTGCAGCGAGTTAG
AGGTGGTTCCATGTGGAATAGTGGGATTGGTACGTGATGGTTGGATGGGGGAATGATGT
GTGTATGGGTGAGGAAAATCGGAGGTTGCGGTGCGAGCGGCAGTAG
>PP968739
GAAGAGCTTCGGCTCTGAATATCTATGGCTAGATAAAGTACAAGTCGTAACAAGGTTTC
AGTTGGAGAACCAGCTGAAGGGTCATTTTCAGTTTTTGGGGTGTGGGTATCGGAATGT
GTGGTAGGTGATGTGTGTGTGTATGGGGGATGCCGAGGGGACCAGCGGTGCGGTGGTG
TGTGCAGGCGTGAGAGTGTATCTGCAAGGGTGAGGGATGTGGGTGCAGCGAGTTAGA
GGTGGTTCCATGTGGAATAGTGGGATTGGTACGTGATGGTTGGATGGGGGAATGATGTG
TGTATGGGTGAGGAAAATCGGAGGTTGCGGTGCGAGCGGCAGTA
Wild population:
>PX579110
CAAGTGATCCTGTATTAGGGATATTTCCAAATACACCTCTTGATGGCACCCTACTGCCGC
TCGCACCGCAACCTCCGATTTTCCTCACCCATACACACATCATTCCCCCATCCAACCATC
ACGTACCAATCACACTATTCCACATGGAACCACCTCTAACTCGCTGCACCCACATCCCT
CACACTTGCAGATACACTCTCACGCCTGCACACACCACCGCACCGCTGGTCCCCTCGG
CATCCCCCATACACACACACATCACCTACCACACATTCCGATACCCACACCCCAAAAAC
TGAAAATGATCCTTCAGCTGGTTCTCCAACTGAAACCTTGTTACGACTTGTACTTTATCT
AGCCATAGATATTCAGAGCCGAAGCTCTTCATCCCTATG

5. Conclusion

Although the original uncropped gel images are unavailable, all positive samples were independently validated through bidirectional Sanger sequencing and publicly archived in GenBank.

These sequence data provide molecular confirmation supporting the PCR findings and genotype assignments reported in the manuscript.

We respectfully request that the editorial office and reviewers consider the attached sequencing-based evidence and supplementary documentation in support of the present study.
